# A new nursing pattern based on ERAS concept for patients with lumbar degenerative diseases treated with OLIF surgery: A retrospective study

**DOI:** 10.3389/fsurg.2023.1121807

**Published:** 2023-04-06

**Authors:** Hai-rong Lu, Ao Yang, Xu Li, Meng-zi He, Jia-yuan Sun

**Affiliations:** Department of Spine Surgery, The Third Hospital of Hebei Medical University, Shijiazhuang, China

**Keywords:** ERAS application®, OLIF = oblique lateral interbody fusion, ERAS (enhanced recovery after surgery), spine surg, lumbar degeneration diseases, surgical outcome

## Abstract

**Objective:**

The purpose of this study was to introduce enhanced recovery after surgery (ERAS) concept into patients with lumbar degenerative diseases who were treated with oblique lumbar interbody fusion (OLIF), and to assess whether it could increase clinical efficacy, reduce perioperative complications, shorten length of hospital stay (LHS), decrease readmission rate, and improve patient satisfaction.

**Methods:**

The study included patients with lumbar degenerative diseases (LDDs) who underwent OLIF between July 2017 and October 2018 (non-ERAS group), and between November 2018 and July 2020 (ERAS group). The two groups were compared according to the demographic and clinical characteristics.

**Results:**

There was no significant difference in descriptive characteristics and concomitant diseases between the two groups. The preoperative Oswestry disability index (ODI) score (*P* = 0.191), lumbar visual analogue scale (VAS) score (*P* = 0.470), and leg VAS score (*P* = 0.657) did not significantly different. Most of the ERAS measures were also well implemented after surgery, except for early delivery (74.2%), early catheter removal (63.9%), and multimodal analgesia (80.6%). The LHS in the ERAS group was significantly shorter than that in the non-ERAS group (*P* = 0.004). Besides, Hamilton Anxiety Rating Scale (HAMA) score at 3 days after surgery showed a significant difference between the two groups (*P* = 0.019). The patient satisfaction in ERAS group was significantly higher than that in the non-ERAS group (*P* = 0.001).

**Conclusion:**

The new nursing pattern combined with ERAS in patients with LDDs who underwent OLIF did not improve the short-term prognosis of surgery, while it could effectively reduce postoperative complications, shorten the LHS, and improve patient satisfaction, and did not lead to additional adverse events.

## 1. Introduction

Lumbar degenerative diseases (LDDs) are the most common diseases in spinal surgery, which affect the quality of life of patients and also cause a very serious economic burden on society. With the aggravation of population aging, global economic losses are also increasing year by year (1). For patients with severe symptoms, lumbar interbody fusion is mainly necessary in clinical practice. Traditional lumbar interbody fusion includes posterior lumbar interbody fusion (PLIF), transforaminal lumbar interbody fusion (TLIF), etc. However, in recent years, minimally invasive surgery has noticeably attracted surgeons' attention. Compared with traditional surgery, minimally invasive surgery possesses the advantages of smaller incision, less soft tissue damage, and shorter postoperative recovery time (2).

Oblique lumbar interbody fusion (OLIF) was first proposed by Silvestre et al. (3) in 2012, and it has been widely used in recent years. OLIF reaches the lumbar space through the space between the retroperitoneal abdominal vascular sheath and the anterior edge of the psoas major muscle. Compared with the traditional posterior lumbar surgery, OLIF has less interference with the posterior muscles and ligaments, less blood loss, and faster postoperative recovery. It can effectively reduce the incidence of pain syndrome after lumbar surgery (4).

Enhanced recovery after surgery (ERAS) is a nursing concept on the basis of evidence-based medicine, which aims to improve the prognosis of patients, reduce the length of hospital stay (LHS), and optimize patient satisfaction. It has been widely used in several surgical fields, and has exhibited a satisfactory effect (5–7). In addition, previous studies have also proved its application in lumbar surgery. However, the previous application of ERAS in lumbar surgery was mainly limited to posterior surgery, such as traditional PLIF/TLIF (8) or minimally invasive surgery [e.g., percutaneous endoscopic lumbar discectomy (9)], while no study has assessed its effect in OLIF. OLIF is also a minimally invasive surgery, and its concept is more consistent with that of ERAS. The present study aimed to introduce ERAS concept into patients with LDDs who were treated with OLIF, and to indicate whether it can increase clinical efficacy, reduce the incidence of perioperative complications, shorten LHS, decrease readmission rate, and improve patient satisfaction.

## 2. Materials and methods

### 2.1. Patients

The study included patients with LDDs who underwent OLIF between July 2017 and October 2018 (non-ERAS group), and between November 2018 and July 2020 (ERAS group). The inclusion criteria were as follows: (1) Patients who were diagnosed with LDDs and underwent stand-along OLIF; (2) Availability of complete clinical data; (3) The last follow-up was longer than 6 weeks. The exclusion criteria were as follows: (1) Combination with other spinal cord-related diseases; (2) Trauma, inflammation, infection, and tumor involvement of the spine, as well as those cases who planned for a revision of a previous fusion. A total of 103 patients were included in the study, of whom there were 41 patients in the ERAS group, and 62 patients in the non-ERAS group. Discharge of patients in our study was based on their clinical status. Both groups were cured by the same surgical team. Diagnosis of LDDs and selection of surgical method was performed by three spinal orthopedic specialists based on clinical symptoms and imaging findings.

Demographic data included age, gender, and history of smoking and drinking. Comorbidities included hypertension, heart disease, diabetes, osteoporosis, stomach problem, bowel or intestinal problem, and psychological symptoms. Other relevant data included Oswestry disability index (ODI) score and visual analogue scale (VAS) score. The surgical data were reviewed to record the operation time and intraoperative blood loss. Outcome measures included LHS, Hamilton Anxiety Rating Scale (HAMA) score at 3 days after surgery, mean postoperative VAS score, patient satisfaction at discharge and at 6 weeks after discharge, and 6-week readmission rate.

Patients' nursing satisfaction score was based on their satisfaction scores from a questionnaire provided by the Spine Surgery Department of the Third Hospital of Hebei Medical University, which was divided into 9 items, including respect the patients, listening to the appeal, information of the conditions, waiting time, procedure convenience, catering services, attention information, medication information, and overall experience of hospitalization. The first eight items were scored according to the patient satisfaction, with a maximum of 5 points, and the last item was the patient's overall medical experience, with a maximum of 10 points, with the full score of 50 points. The follow-up staff received unified training and were asked to use standardized interrogation methods without any interference or guidance to patients.

### 2.2. ERAS interventions

ERAS interventions were developed by anesthesiologists, spine surgeons, nutritionists, rehabilitation physicians, internists, and nurses. Through literature review and empirical discussion, reasonable ERAS intervention programs were obtained. This study was approved by the Ethics Committee of the Third Hospital of Hebei Medical University.

Compared with routine lumbar surgical care, the nursing measures for patients undergoing single-level OLIF were improved in the following aspects to better conform to the ERAS concept: (1) The patient was educated again after surgery. (2) Take carbohydrate drinks 2 h before surgery. (3) Warm the liquid to 37°C (98.6°F) using an infusion heater without compromising the effect of the drug. (4) Early postoperative activity. (5) Use analgesics at 2 h before surgery. (6) Use an insulated mattress.

ERAS nursing measures are mainly divided into three aspects: preoperative, intraoperative, and postoperative, which can be presented as follows: (1) patient's education, (2) shortened preoperative fasting, (3) preoperative antibiotics, (4) standard anesthesia regimen, (5) multimodal analgesia, (6) early oral feeding (7) early ambulation, (8) early removal of bladder catheter, (9) antithrombotic prophylaxis, (10) maintenance of patient's body temperature, (11) improvement of the sleep quality of patients, and (12) monitoring of vital signs (Table 2).

### 2.3. Statistical analysis

SPSS 22.0 software (IBM, Armonk, NY, USA) was used for statistical analysis, and statistical level was set to *P* = 0.05. According to normal distribution and homogeneity of variance of data, we used independent-sample *t*-test or Mann-Whitney *U*-test to compare and analyze the data between the two groups. Comparison of patient satisfaction scores was performed by paired *t*-test or Wilcoxon test. Count data were analyzed by *χ*^2^ test.

## 3. Results

A total of 103 patients with LDDs who were treated with OLIF were enrolled in this study, including 42 men and 61 women, with an average age of 58.48 ± 7.87 years. Patients were divided into ERAS group (*n* = 62) and non-ERAS group (*n* = 41) according to whether the perioperative ERAS intervention was applied. Statistical analysis showed that there was no significant difference in descriptive characteristics and concomitant diseases between the two groups. There was no significant difference in preoperative ODI score (*P* = 0.191), lumbar VAS score (*P* = 0.470), and leg VAS score (*P* = 0.657) between the two groups. All surgeries were performed by 3 experienced spine surgeons. There was no significant difference in operation time (*P* = 0.624) and intraoperative blood loss (*P* = 0.312) between the two groups (Table 1).

**Table 1:** Comparison of patients’ demographic and clinical characteristics between ERAS group and Non-ERAS group.

Characteristic	ERAS	Non-ERAS	*P*
Sample size	62	41	
Age (years)	57.27 ± 7.95	60.29 ± 7.58	0.524
Male/female	28/34	14/27	0.309
Smoker	28	10	0.309
Drinker	21	11	0.518
**Comorbidities**			
Hypertension	40	26	0.909
Heart disease	12	11	0.373
Diabetes	14	15	0.122
Osteoporosis	11	7	0.930
Cerebrovascular disease	18	6	0.091
Preoperative ODI, %	67.40 ± 8.40	65.10 ± 9.13	0.191
Preoperative VAS (back)	6.47 ± 1.34	6.66 ± 1.26	0.470
Preoperative VAS (leg)	5.84 ± 1.40	5.71 ± 1.55	0.657
Operation time	94.05 ± 14.10	95.46 ± 14.60	0.624
Intraoperative blood loss	133.71 ± 25.37	128.54 ± 25.16	0.312

In the ERAS group, the performance of each ERAS intervention was assessed (Table 2). It was revealed that preoperative and intraoperative ERAS measures were well implemented (>90%), and most of the ERAS measures were also well implemented after surgery, except for early delivery (74.2%), early catheter removal (63.9%), and multimodal analgesia (80.6%).

**Table 2:** The compliance with the new nursing pattern based on ERAS concept.

Compliance with the ERAS program	
Variable	*n* (%)
**Preoperative ERAS items**	
Patient education	62 (100%)
Carbohydrate drinks intake	60 (96.8%)
Antimicrobial prophylaxis	62 (100%)
Improve patients’ sleep quality	58 (93.5%)
**Intraoperative ERAS items**	
Tranexamic acid	62 (100%)
Using warmed liquid	62 (100%)
Local infiltration analgesia	62 (100%)
**Postoperative ERAS items**	
Vital signs monitoring	62 (100%)
Early ambulation	46 (74.2%)
Early removal of bladder catheter	39 (62.9%)
Early oral feeding	50 (100%)
Multimodal analgesia	58 (80.6%)
Improve patients’ sleep quality	59 (95.2%)
Using pneumatic pump	62 (100%)
Using warmed liquid	62 (100%)
Using temperature adjustable mattress	62 (100%)
Patient education after surgery	62(100%)

No cerebrovascular accident or cardiac arrest was recorded. In the non-ERAS group, there were 1 case of deep vein thrombosis in the lower extremities, 2 cases of cerebrospinal fluid (CSF) leakage, and 1 case of delirium. In the ERAS group, there were 1 case of incision infection and 1 case of CSF leakage. Overall, there was no significant difference in postoperative complications between the two groups. There was no significant difference in VAS score between the two groups on the first postoperative day (1.73 ± 0.93 vs. 1.88 ± 1.12, *P* = 0.455) and the third postoperative day (1.58 ± 1.21 vs. 1.83 ± 1.18, *P* = 0.305). The LHS in the ERAS group was significantly shorter than that in the non-ERAS group (8.39 ± 1.94 vs. 9.49 ± 1.80, *P* = 0.004). Moreover, HAMA at 3 days after surgery showed a significant difference between the two groups (5.34 ± 2.33 vs. 6.61 ± 3.05, *P* = 0.019). There was no significant difference in the readmission rate and mortality rate within 6 weeks between the two groups (Table 3).

**Table 3:** Comparison of patients’ outcomes between ERAS group and Non-ERAS group.

Outcome measure	ERAS	Non-ERAS	*P*
**Complications**			
Cerebrovascular accident	0	0	
Cardiac arrest	0	0	
Deep vein thrombosis	0	1	0.398
Surgical site infection	1	0	0.602
Spinal fluid leakage	1	2	0.562
delirious state	0	1	0.398
LOS	8.39 ± 1.94	9.49 ± 1.80	0.004*
VAS 1 days after surgery	1.73 ± 0.93	1.88 ± 1.12	0.455
VAS 3 days after surgery	1.58 ± 1.21	1.83 ± 1.18	0.305
HAMA 3 days after surgery	5.34 ± 2.33	6.61 ± 3.05	0.019*
6-week readmission	0	1	0.398
6-week mortality	0	0	

LOS, length of stay.

*means *P* <0.05.

**Figure 1:**
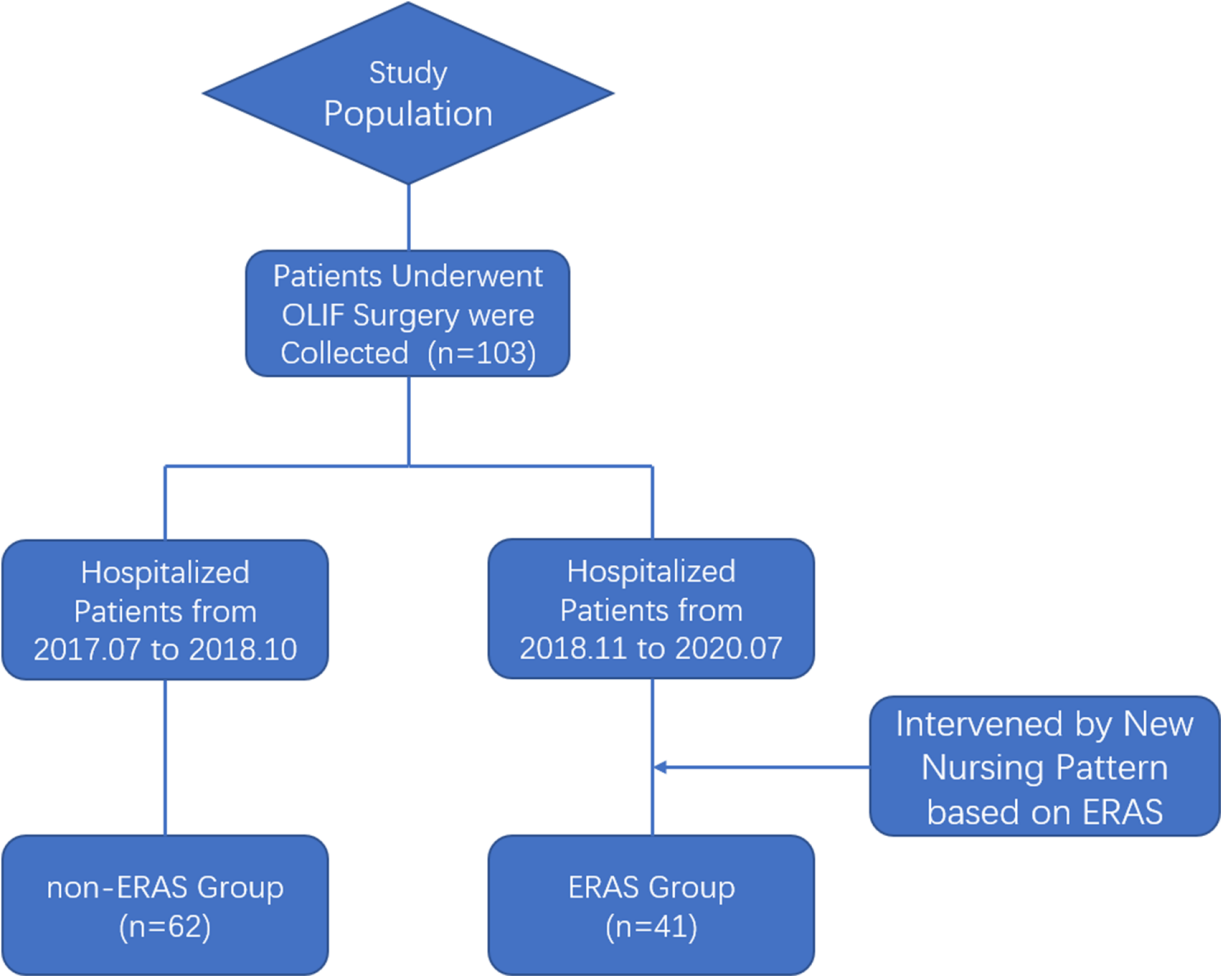
Research flow chart.

Inpatient satisfaction was analyzed and each item was counted. It was found that there were statistically significant differences between the two groups in terms of information of the conditions (*P* = 0.040), waiting time (*P* = 0.004), and overall experience of hospitalization (*P* = 0.026). It is noteworthy that the two groups also showed significant differences in total scores (*P* = 0.001) (Table 4, Figure 1).

**Figure 2:**
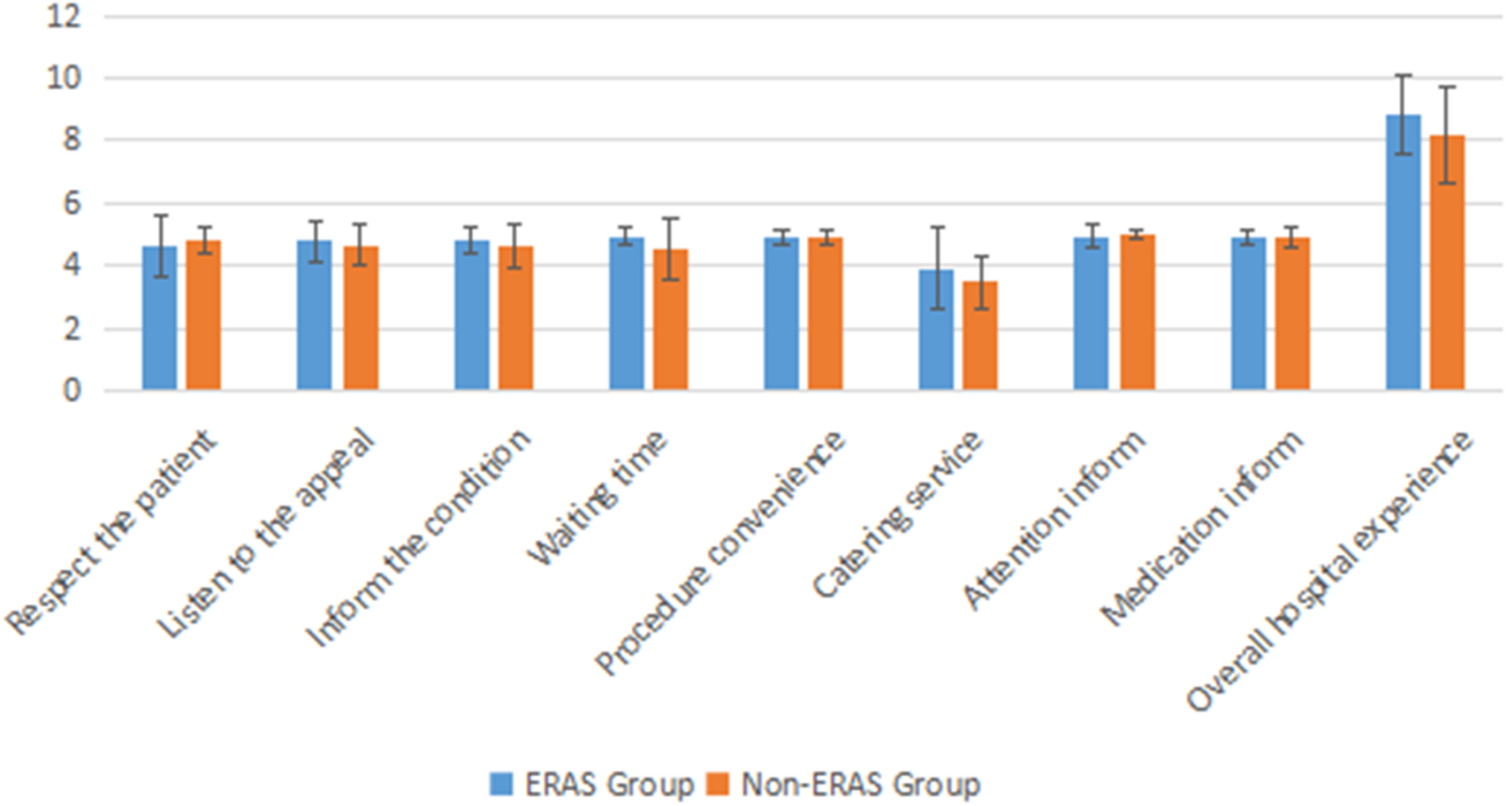
Comparison of patient satisfaction scores between ERAS group and Non-ERAS group.

**Table 4:** Comparison of patient satisfaction score between ERAS group and Non-ERAS group.

	ERAS (*n* = 62)	Non-ERAS (*n* = 41)	*P*
Respect the patient	4.61 ± 0.96	4.83 ± 0.44	0.182
Listen to the appeal	4.79 ± 0.66	4.66 ± 0.66	0.321
Inform the condition	4.84 ± 0.41	4.61 ± 0.70	0.040*
Waiting time	4.95 ± 0.28	4.56 ± 1.00	0.004*
Procedure convenience	4.94 ± 0.25	4.93 ± 0.26	0.866
Catering service	3.90 ± 1.31	3.51 ± 0.84	0.094
Attention's inform	4.92 ± 0.38	4.98 ± 0.16	0.366
Medication's inform	4.92 ± 0.27	4.93 ± 0.35	0.903
Overall experience of hospitalization	8.85 ± 1.27	8.20 ± 1.55	0.026*
Total points	45.20 ± 2.52	46.73 ± 2.11	0.001*

*means *P* <0.05.

## 4. Discussion

In recent years, with the development of surgical techniques, minimally invasive surgery has become the mainstream surgery in various departments. Compared with the traditional posterior PLIF/TLIF surgery, the OLIF can significantly reduce the damage to the posterior muscle and soft tissue (10). However, with the extensive development of OLIF, its complications are also increasing, leading to the prolongation of LHS and the increase of the cost of hospitalization (11), which is not consistent with the minimally invasive concept. Therefore, this study aimed to provide a better perioperative care for patients with LDDs who received single-level OLIF using ERAS concept, so as to reduce the incidence of complications, shorten the LHS, and improve the patient satisfaction.

In this study, there was no significant difference in demographic characteristics between the ERAS group and the non-ERAS group, and patients in the ERAS group performed our care measures well. In comparison, the three implementation degrees of early ambulation, early removal of bladder catheter tissue, and multimodal analgesia were slightly worse. This may be because LDDs are more common in elderly patients (12), and braking may affect the patient's muscle tissue, leading to neck pain, dizziness, and other symptoms (13). However, the muscle tissue of elderly patients is degraded, thus, they need to adapt to the normal ground for a period of time, resulting in poor execution degree of early ambulation. A study has proved (14) that urinary incontinence has a high incidence in elderly women, while elderly men mainly have benign prostatic hyperplasia (15), which may lead to the symptoms of poor urine control, so that the bladder catheter cannot be removed at the early-stage. Moreover, elderly patients are often accompanied with the risk of cardiovascular diseases and gastrointestinal diseases (16), and the use of non-steroidal anti-inflammatory drugs (NSAIDs) may increase the risk of cardiovascular diseases and gastric bleeding (17), which may remarkably limit the use of analgesic drugs, thereby leading to the failure of multimodal analgesia.

In the postoperative results, there was no significant difference between surgical complications and postoperative VAS score, which was inconsistent with results of a previous study (18). However, our data showed that the mean VAS score of patients in ERAS group at 1 and 3 days after surgery was lower than that in non-ERAS group, thus, we speculated that this might be related to the small sample size of this study. However, this study found that the HAMA score of patients in the ERAS group was significantly lower than that in the non-ERAS group at 3 days after surgery, which proved that the mental state of patients in the ERAS group was better than that in the non-ERAS group. This could be related to the fact that we re-educated patients after surgery and administered analgesics before bedtime. A review study conducted by Friedrich Sabine (19) proved that preoperative anxiety can lead to poor postoperative pain control and increase the incidence of complications, thus, it is necessary to tailor counseling programs for different patients to alleviate anxiety symptoms. However, in clinical tests, we found that although patients were educated in surgery complications, such as preoperative reaction to anesthesia, postoperative wound pain, lower limb transient weakness, several patients will still be in a state of anxiety when the complications appear after the surgery, thus, we educated patients again after the surgery, and achieved a satisfactory effect. The VAS score in the ERAS group was also lower than that in the non-ERAS group at 3 days after surgery, although there was no significant difference between the two groups. The difference in HAMA score after surgery could also be related to the use of analgesics before going to bed. Gulsen et al. (20) conducted a study on 130 employees on campus, and showed that the quality of sleep was associated with anxiety. The new nursing plan in this study added analgesics to patients at 2 h before going to bed after surgery. It not only conforms to the concept of multimodal analgesia in ERAS, but also can improve the sleep quality of patients, so as to relieve anxiety and further improve the patient satisfaction in hospitals.

One of the complications of OLIF patients is vascular injury (21), and the incision is in the abdomen, thus, traditional gastrointestinal care, such as hot compress and massage, is very limited, and patients are prone to postoperative abdominal distension, nausea, vomiting, and other adverse reactions. However, in the traditional nursing, the time of the ground is late, which further aggravates the gastrointestinal symptoms of patients, and greatly increases the severity of pain in patients, leading to a poor prognosis and the decline of patient satisfaction. Traditional fasting for 8 h before surgery and eating for 1 day after surgery may lead to insulin resistance and metabolic stress, and metabolic stress may also increase the incidence of postoperative complications (22, 23). In ERAS concept, early ambulation is an important item, which can reduce the incidence of deep vein thrombosis, promote exhaust, shorten the LHS, and reduce the mortality (24, 25). Shortening fasting and early postoperative feeding can improve the metabolic status of patients, enhance resistance, and help patients recover their appetite and shorten the LHS (26). The results of this study also supported the above-mentioned findings. In our study, LOS was significantly lower in the ERAS group than that in the non-ERAS group.

In the postoperative patient satisfaction, the total satisfaction score of patients in the ERAS group was significantly higher than that in the non-ERAS group, which was in line with the patient-centered concept of ERAS. We also compared each item separately, and patients in the ERAS group were significantly higher than those in the non-FST group in terms of inform the condition, waiting time, and overall experience of hospitalization (Figure 2). This could be related to our post-operative re-education. It is noteworthy that, according to our clinical data, there was no significant difference in the waiting time (such as waiting time for examination, calling medical staff, waiting time for medical staff to arrive, etc.) between the two groups, while the patient satisfaction in the ERAS group was higher than that in the non-ERAS group, which could be related to the lower LHS in the ERAS group. A study showed that using the heating liquid, a heating mattress can avoid perioperative hypothermia, while for prognosis with the operation, there is no additional benefit (27). We express that even if there was no additional benefit for surgical analysis, it can improve patient satisfaction to the hospital. Measures, such as shortening the fasting time and early postoperative feeding, can not only promote the patient's appetite, but also improve the patient's hospitalization experience.

This study has the following limitations: (1) This is a single-center, retrospective study with a limited sample size. In the future, additional multicenter, prospective, large-scale studies will be required to verify the results of this study. (2) The overall follow-up time of this study was not long enough. The surgical approach for patients undergoing OLIF was different from the traditional posterior approach, thus, the postoperative rehabilitation of patients undergoing OLIF should also be unique, which requires long-term follow-up to obtain the results. (3) Due to policy changes, this study did not analyze the impact of nursing model combined with ERAS concept on patients' clinical costs. (4) The survey of patient satisfaction did not indicate an appropriate rating scale, thus, it was impossible to determine in more detailed the specific aspects of the improvement of patient satisfaction.

## 5. Conclusions

The new nursing pattern combined with ERAS in patients undergoing OLIF did not improve the short-term prognosis of surgery, while it could effectively reduce postoperative anxiety, shorten the LHS, and improve patient satisfaction, and it did not lead to additional adverse events.

## Data Availability

The raw data supporting the conclusions of this article will be made available by the authors, without undue reservation.
